# Blood Rheological Characterization of β-Thalassemia Trait and Iron Deficiency Anemia Using Front Microrheometry

**DOI:** 10.3389/fphys.2021.761411

**Published:** 2021-10-21

**Authors:** Lourdes Méndez-Mora, Maria Cabello-Fusarés, Josep Ferré-Torres, Carla Riera-Llobet, Elena Krishnevskaya, Claudia Trejo-Soto, Salvador Payán-Pernía, Inés Hernández-Rodríguez, Cristian Morales-Indiano, Tomas Alarcón, Joan-Lluis Vives-Corrons, Aurora Hernandez-Machado

**Affiliations:** ^1^Department of Condensed Matter Physics, University of Barcelona, Barcelona, Spain; ^2^Centre de Recerca Matemàtica, Barcelona, Spain; ^3^Red Cell Pathology and Hematopoietic Disorders (Rare Anemias) Unit, Josep Carreras Leukaemia Research Institute, Badalona, Spain; ^4^Instituto de Física, Pontificia Universidad Católica de Valparaiso, Valparaiso, Chile; ^5^Red Blood Cell Disorders Unit, Hematology Department, Hospital Universitario Virgen del Rocío, Instituto de Biomedicina de Sevilla (IBIS/CSIC), Seville, Spain; ^6^Hematology Service, Institut Català d’Oncologia, Germans Trias i Pujol University Hospital, Badalona, Spain; ^7^Laboratory Medicine Department, Laboratori Clínic Metropolitana Nord, Hospital Universitari Germans Trias i Pujol, Badalona, Spain; ^8^Institució Catalana de Recerca i Estudis Avançats, Barcelona, Spain; ^9^Departament de Matemàtiques, Universitat Autónoma de Barcelona, Bellaterra, Spain; ^10^Institute of Nanoscience and Nanotechnology, University of Barcelona, Barcelona, Spain

**Keywords:** beta-thalassemia trait, iron deficiency (anemia), anemia, hemorheology, rheology, microfluidics, blood rheology, microrheometer

## Abstract

The purpose of this work is to develop a hematocrit-independent method for the detection of beta-thalassemia trait (β-TT) and iron deficiency anemia (IDA), through the rheological characterization of whole blood samples from different donors. The results obtained herein are the basis for the development of a front microrheometry point-of-care device for the diagnosis and clinical follow-up of β-TT patients suffering hematological diseases and alterations in the morphology of the red blood cell (RBC). The viscosity is calculated as a function of the mean front velocity by detecting the sample fluid-air interface advancing through a microfluidic channel. Different viscosity curves are obtained for healthy donors, β-TT and IDA samples. A mathematical model is introduced to compare samples of distinct hematocrit, classifying the viscosity curve patterns with respect to the health condition of blood. The viscosity of the fluid at certain shear rate values varies depending on several RBC factors such as shape and size, hemoglobin (Hb) content, membrane rigidity and hematocrit concentration. Blood and plasma from healthy donors are used as reference. To validate their potential clinical value as a diagnostic tool, the viscosity results are compared to those obtained by the gold-standard method for RBC deformability evaluation, the Laser-Optical Rotational Red Cell Analyzer (LoRRCA).

## Introduction

Blood is a biological fluid composed of red blood cells (RBCs), white blood cells (WBC), and platelets suspended in plasma, a Newtonian fluid containing organic molecules, proteins and salts (Baskurt and Meiselman, 2003). The viscosity of plasma is constant; it does not depend on the pressure that is applied onto it (Késmárky et al., 2008). Whole blood is a non-Newtonian shear-thinning fluid, and its viscosity decreases when an increasing pressure is applied. WBCs and platelets can affect blood rheology but, under normal conditions, RBCs represent most of the cellular components and make the biggest contribution to viscosity (Pop et al., 2002). As a result, the viscosity of blood depends mostly on the ability of RBCs to deform under circulation flow (Baskurt and Meiselman, 2003; Cokelet and Meiselman, 2007; Mehri et al., 2018).

Red blood cells have a characteristic discoid shape with a biconcave profile, under normal physiological conditions; the well-known discocyte shape (Diez-Silva et al., 2010). This shape combines a large area to volume ratio, which is an important factor to maximize oxygen diffusion. The erythrocytes have a unique ability to deform and pass through small capillaries before rapidly recovering their initial shape. The deformation capacity of RBCs is mainly due to three factors; their large area to volume ratio; the viscosity of the intracellular fluid that is dominated by the presence of hemoglobin and the viscoelastic properties of the cell membrane (Lázaro et al., 2013; Tomaiuolo, 2014). However, the deformability of RBCs is impaired in some pathological conditions as a result of defects in cell membrane skeletal architecture (Faustino et al., 2019; Hymel et al., 2020), erythrocyte aging (Diez-Silva et al., 2010; Simmonds et al., 2013), and mechanical damage (Rico et al., 2018).

β-thalassemia syndromes are a heterogeneous group of genetic conditions characterized by reduced or abolished synthesis of the hemoglobin subunit beta (hemoglobin beta chain). The β-thalassemia carrier state is also referred as heterozygous β-thalassemia or β-thalassemia trait (β-TT) (Galanello and Origa, 2010). When β-TT is present, RBCs show rheological abnormalities (Berga et al., 1989). The RBC membrane can be affected due to a selective interaction between the α-chain excess and the erythrocyte membrane cytoskeleton (Rachmilewitz and Kahane, 1980), while other alterations are caused by hemoglobin denaturalization (Fiorelli et al., 1996). In addition, microcytosis, or decreased mean corpuscular volume (MCV) of RBCs, is present in β-TT carriers. Previous works suggest that the decreased RBC deformability is probably due to microcytosis (Rasia et al., 1986; Iborra et al., 2003). More recently, by comparing two non-healthy sets of samples, of iron deficiency anemia (IDA) (DeLoughery, 2017) and β-thalassemia trait, a study based on ektacytometry showed two distinct deformation patterns, indicating that the observed decrease in the deformability is not only due to microcytosis (Payán-Pernía et al., 2021). Osmotic gradient ektacytometry measures RBC deformability under defined shear stress as a function of suspending medium osmolarity (Llaudet-Planas et al., 2018). IDA and β-TT are two very common causes of microcytic hypochromic anemia in the Mediterranean region (Galanello and Origa, 2010).

Alterations in the deformation capacity of RBCs can produce changes in the non-linear rheological behavior of the whole blood; these alterations are potentially detectable via a rheometry study. Rheological measurements are usually performed with rotational rheometers, a type of shear rheometer (Macosko, 1994). However, this type of equipment is expensive, requires a considerable laboratory space and must be operated by trained personnel. Moreover, the measurements take several minutes and need a large sample amount. For these reasons, there is a demand for Point-of-care (PoC) devices for analyzing the rheological properties of whole blood. To be efficient and cost-effective, many portable PoC devices employ microfluidic devices that generally consist of low-cost microfluidic chips, that are easy to fabricate and are made of glass, polydimethylsiloxane (PDMS) and other polymeric materials that are biocompatible and widely available.

The microfluidic approach permits to obtain a high precision measurement of blood conditions in motion with only one drop of blood, in a time and cost-effective way in comparison to rotational rheometers (Davies and Stokes, 2008; Pipe and McKinley, 2009; Choi and Park, 2010; Galindo-Rosales et al., 2012). Many applications that aim at analyzing the properties of the blood through its rheological characterization, employ microfluidic devices that measure the behavior of blood as a function of the shear rate (Srivastava and Burns, 2006; Fedosov et al., 2011; Tomaiuolo, 2014; Rico et al., 2018; Rodriguez-Villarreal et al., 2020). In addition, some microfluidic applications permit the observation of the flow of blood under high confinement conditions (Davies and Stokes, 2008; Bartolo and Aarts, 2012; Lázaro et al., 2014). PoC diagnostics, including microfluidic tools, are very promising for early detection of different diseases, and for the monitoring of health conditions.

In this work, a PoC front-microrheometer with electronic detection is used to characterize blood samples from different donors. The viscosity value as a function of the shear rate of the blood front is measured, details on the mathematical model developed can be found in previous works (Trejo-Soto et al., 2017, Trejo-Soto et al., 2018; Méndez-Mora et al., 2021). Three types of samples are analyzed: control samples from healthy donors, and blood samples from IDA and β-TT patients. Due to the morphological and membrane differences of the RBCs in the three groups, it is expected to obtain a distinctive viscosity pattern for each group. As it is expected that RBCs volume has an impact on blood viscosity, and the microcytosis is present in IDA and β-TT blood donors, the samples are classified in terms of the MVC of their RBCs, ensuring that microcytosis does not mask other rheological effects.

The main purpose of this study is to demonstrate that the micro-rheometer developed can differentiate distinct hematological pathologies, regardless their hematocrit percentage. This is done by the differentiation, through whole-blood viscosity, between two very common conditions that affect RBCs, IDA, and β-TT, beyond the effect of the low MCV that characterizes both of them, and after hematocrit normalization. The research represents and advancement in the development of PoC devices for the diagnosis of anemia-related diseases. This is of global concern, since information about the precise world distribution and frequency of the inherited and acquired hemoglobin disorders is still limited (Williams and Weatherall, 2012). It is also an issue of particular interest in the developing countries, where anemia is more prevalent (Kassebaum et al., 2016).

## Materials and Methods

### Mathematical Model

The method used here to analyze the fluid flow consists of the study of the fluid-air interface advancement. Inside a microfluidic channel, the front velocity $\dot{h}$, is the change in position of the fluid, *h*(*t*) through time along the microfluidic channel. The term *h*(*t*) is the average position of the front $h\left(t\right)=\frac{1}{N}\sum_{j=1}^{N}h_{j}\left(t\right)$, where *h*_*j*_(*t*) is the fluid front position. Shear rate, $\dot{\gamma }$ is the normalization of mean front velocity $\left(\dot{h}\right)$ according to the depth of the microchannel, *b*:


(1) $$\dot{\gamma }=\frac{\dot{\text{h}}}{\text{b}}$$

According to the Ostwald-De Waele Power-Law model for fluids, the viscosity can be expressed as a function of shear rate:


(2) $$\eta (\gamma )=\text{m}{\dot{\gamma }}^{n-1}$$

where *m* is a prefactor obtained experimentally, n is a constant that depends on the studied fluid and defines the nature of its viscosity, being n = 1 for Newtonian fluids. To remove the effects on viscosity caused by distinct hematocrit levels in different samples, the first step is to calculate the effective viscosity, (η_eff_). This is the viscosity of blood relative to the viscosity of plasma. It consists of establishing a relation between the pressure versus shear rate fit for plasma and the sample that needs to be normalized (Trejo-Soto and Hernández-Machado, 2021). This is expressed as follows:


(3) $$\eta_{\mathrm{eff}}=\frac{{\dot{\gamma }}_{\mathrm{Plasma}}\Delta {\text{P}}_{\mathrm{Sample}}}{{\dot{\gamma }}_{\mathrm{Sample}}\Delta {\text{P}}_{\mathrm{Plasma}}}$$

where ΔP_Sample_ and ΔP_Plasma_ are the pressure inside the microfluidic system for the blood sample and its plasma, respectively. Analogously, ${\dot{\gamma }}_{\text{Sample}}$ and ${\dot{\gamma }}_{\text{Plasma}}$ are the shear rates of the blood and plasma samples, respectively. Finally, (η_eff_) is normalized with respect to the maximum hematocrit level of the studied samples. The hematocrit normalized viscosity (η_hct_), is calculated using the following equation:


(4) $$\eta_{\mathrm{hct}}=1+\left(\eta_{\mathrm{eff}}-1\right)\frac{\phi_{\max}}{\phi_{\mathrm{sample}}}$$

where ϕ_*max*_ is the maximum hematocrit with respect to which the different samples need to be normalized. ϕ_sample_ is the hematocrit of the sample being normalized.

### Experimental Method

The experimental setup used consists of a microfluidic channel with 24 gold electrodes, a pumping source, and a computer terminal (Méndez-Mora et al., 2021). A series of shear rates is obtained by applying a set of pressures on the sample fluid. The fluid front advancement is detected in a fast and accurate manner by the gold electrodes printed beneath the microfluidic channel. The microfluidic chips made of polydimethylsiloxane (PDMS) attached to glass substrates by oxygen plasma bonding. The microfluidic channel and the electrodes have been fabricated using lithography techniques (Qin et al., 2010). The distance between electrodes is 350 μm. There are four groups of electrodes, and each has six pairs of electrodes. The distance between each group of electrodes is 8.5 mm. The channel width is ω = 1000 μm height b = 300 μm and the length from inlet to outlet is l_c_ = 4cm. PDMS, glass, and Tygon tubes are biocompatible.

**FIGURE 1:**
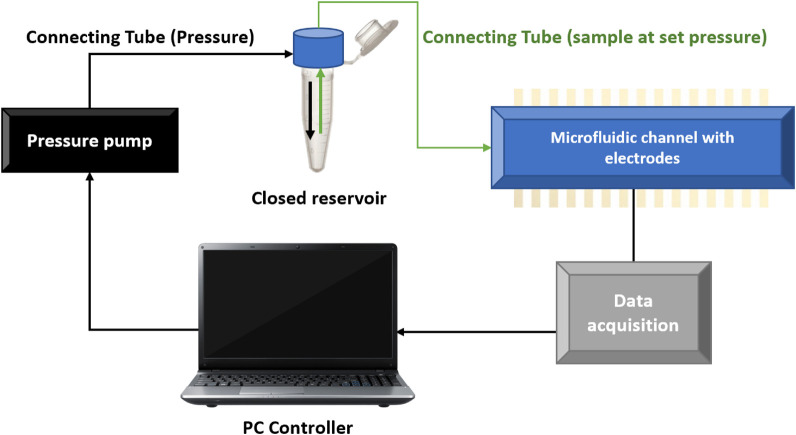
Schematics of the microfluidic setup. A micropump is used as a pressure source. The pump is connected to the reservoir with the fluid sample using a tube. The fluid is flowing through the microchannel. The microfluidic consumable is made of PDMS sealed on a glass substrate with electrodes.

Using a tube of an internal diameter of 254 μm and length 20cm the pump is connected to a closed reservoir holding the fluid, and this communicates the reservoir with the microchannel. The pump is easy to control through the computer, using a simple graphic interface. Different pressures for the Fluika Pump are set to run the experiment, from 1,000 to 5,000 Pa The electrodes in the microfluidic channel are connected to a data acquisition system by a set of contact pins. As soon as the pressure starts being applied, the electronic reading is activated thanks to the controller of a myRIO National Instruments card. This tool communicates the fluidic pump and the electronic reading pins, through the computer. The sample fluid comes out of the reservoir at the set pressure and advances through the channel. As the fluid enters the microchannel and contacts the electrodes, it acts as a switch and sets the time required for the fluid front to reach each electrode pair. Using the time data and the distance between electrodes, it is possible to calculate the fluid front velocity. All these tests are performed at room temperature (∼24°C). The way these microfluidic elements are connected is displayed in Figure 1.

The experimental setup is comprised of a pumping source that connects to a fluid reservoir. The sample stored in the reservoir is pushed out through a connecting tube of radius r, and length l_t_. The tube transporting is connected into the microfluidic channel of rectangular cross-section, of width ω and height *b*. The pressures acting on the system depicted in Figure 1, relate to each other as:


(5) $$\Delta P={\text{P}}_{p}+{\text{P}}_{H}-\Delta {\text{P}}_{t}-{\text{P}}_{\mathrm{cap}}$$

Here ΔP, the pressure drop inside the microchannel, is the summation of all the pressures involved in the experiment. The set pressure P_p_, is the pressure coming from the pumping source. The hydrostatic pressure P_H_ is the product of the density ρ, gravity g, and height of the fluid with respect to the channel height H; expressed as P_H_ = ρgH. The pressure drop represented by the tube connection is ΔP_t_. The capillary pressure, P_cap_ is defined as the resistance that the hydrophobic channel walls oppose to the fluid advancement. P_cap_ is dependent on the contact angle and the surface tension of the fluid, τ.

Thanks to the mass conservation principle, the flow inside the system is $\text{Q}={\text{Q}}_{\text{t}}\to \omega \text{b}\dot{\text{h}}=\pi r^{2}{\text{v}}_{\text{t}}$, where Q_t_ and v_t_, are the flow and velocity inside the tube, respectively, and Q is the flow inside the channel (Méndez-Mora et al., 2021). The relation ${\text{v}}_{\text{t}}=\frac{\dot{\text{h}}\omega \text{b}}{\pi r^{2}}$ is valid for the coupled system and ΔP_t_ can be written as:


(6) $$\Delta {\text{P}}_{t}=\text{m}2{\text{l}}_{t}{\left(\frac{\omega \text{b}}{\pi {\text{r}}^{2}}\right)}^{n}\left(\frac{1}{{\text{r}}^{1+n}}\right){\left(\frac{1}{\text{n}}+3\right)}^{n}{\dot{\text{h}}}^{n}$$

Now, the effective pressure inside the system can be calculated as:


(7) $$\begin{array}{c} {\text{P}}_{\mathrm{eff}}=\text{m}2l_{t}{\left(\frac{\omega \text{b}}{\pi {\text{r}}^{2}}\right)}^{n}\left(\frac{1}{{\text{r}}^{1+n}}\right){\left(\frac{1}{\text{n}}+3\right)}^{n}{\dot{\text{h}}}^{n}\\ \;=\text{m}2{\text{l}}_{t}{\left(\frac{\omega {\text{b}}^{2}}{\pi {\text{r}}^{2}}\right)}^{n}\left(\frac{1}{{\text{r}}^{1+n}}\right){\left(\frac{1}{\text{n}}+3\right)}^{n}{\dot{\gamma }}^{n} \end{array}$$

The mean front velocity $\dot{\text{h}}$, at each set pressure is experimentally obtained. By calculating velocity values and the channel dimensions, shear rate values are obtained. By fitting a curve for the relation between *P*_*eff*_ and $\dot{\gamma }$, the next relation is obtained:


(8) $${\text{P}}_{\mathrm{eff}}=\text{K}\left(m,n\right){\dot{\gamma }}^{n-1}$$

All the independent variables are grouped in K, which depends on the fluid properties (*m* and *n*) and the geometrical parameters of the system (ω, *b*, *l*_*t*_, *r*),


(9) $$\text{K}\left(m,n\right)=\text{m}2{\text{l}}_{t}{\left(\frac{\omega {\text{b}}^{2}}{\pi r^{2}}\right)}^{n}\left(\frac{1}{{\text{r}}^{1+n}}\right){\left(\frac{1}{\text{n}}+3\right)}^{n}$$

### Patients and Sample Preparation

A total of five patients with IDA, 15 patients with β-TT, and 10 normal controls (healthy individuals) were included in the study. The ages range for patients was from 18 to 95 years old. None of the patients had been transfused in the previous months. IDA was considered present when serum ferritin (SF) was <15 μg/L, or when SF was <30 μg/L, and transferrin saturation index (TSI) was <20%. IDA is present when (Hb < 12.0 g/dL in non-pregnant adult women and Hb < 13.0 g/dL in adult men). β-TT was diagnosed if mild microcytic anemia was present in absence of iron deficiency and the Hb analysis showed an increase in HbA2 (>3.5%) with or without an increase in Hb F (if present, <5%). All subjects in the control group had normal Hb levels, RBC indices, and iron parameters.

Samples were collected in ethylenediaminetetraacetic acid (EDTA) anticoagulant tubes during the course of routine analysis, shipped and stored at a temperature of 4°C, and processed within 24 h after the extraction. Complete blood count (CBC) parameters were assessed on a Sysmex XN-10 (Sysmex Corporation) analyzer. Total iron-binding capacity (TIBC), and serum ferritin (SF) were measured on Architect ci16000 System (Abbott Laboratories). Serum iron (SI), and (TIBC) by enzymatic methods, and SF by a two-step chemiluminescent micro-particle immunoassay (CMIA). The (TSI) was calculated as the ratio of iron to TIBC. Hb analysis was performed using high-performance liquid chromatography (HPLC) on a D-10 Dual A2/F/A1c (Bio-Rad Laboratories) or capillary electrophoresis on a Capillaris-2 Flex Piercing (Sebia). The use of the samples was authorized by the Bioethics Committee of the University of Barcelona (IRB 00003099) and the Ethics Committee of IJC [Comité Ético de Investigación Clínica (CEIC)].

Samples with different hematocrit concentrations were manually prepared by properly mixing plasma and RBC in the desired ratio. To obtain plasma, whole blood samples were centrifuged at 2,500 rpm for 5 min. The plasma that rests at the top is collected with a pipette. The different hematocrit concentrations hct (%) are prepared by taking plasma previously separated from whole blood and then adding to it the desired volume of RBCs corresponding to each hematocrit percentage. The sample volume prepared for each individual experiment is 500 μL.

## Results and Discussion

### Blood Viscosity Normalization by Hematocrit

**FIGURE 2:**
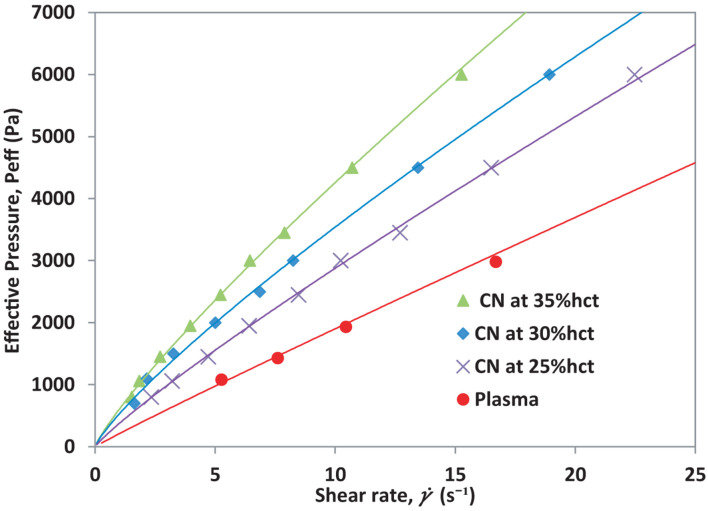
Effective pressure vs. shear rate for blood from a healthy donor at different hematocrit levels.

The hematocrit value in blood samples has a high impact on blood viscosity differences between different patients with the same blood conditions (Baskurt and Meiselman, 2003). Heterogeneity across patients regarding blood viscosity is expected to be present, mostly due to variations in hematocrit, hct (%). To observe differences in viscosity caused by blood abnormalities and be able to compare the blood viscosity curve of different patients, blood viscosity needs to be normalized to account for variations in hematocrit. To validate the normalization procedure, we first take a control sample (CN) from a healthy donor and prepare three different hct (%): 35%, 30%, and 25%. Viscosity is calculated for each hematocrit using Eq. 2. By using Eqs. 3, 4, the effects of hematocrit are removed. The effective pressure, P_eff_ as a function of the shear rate, $\dot{\gamma }$ for each prepared hematocrit and plasma are presented in Figure 2.

**FIGURE 3:**
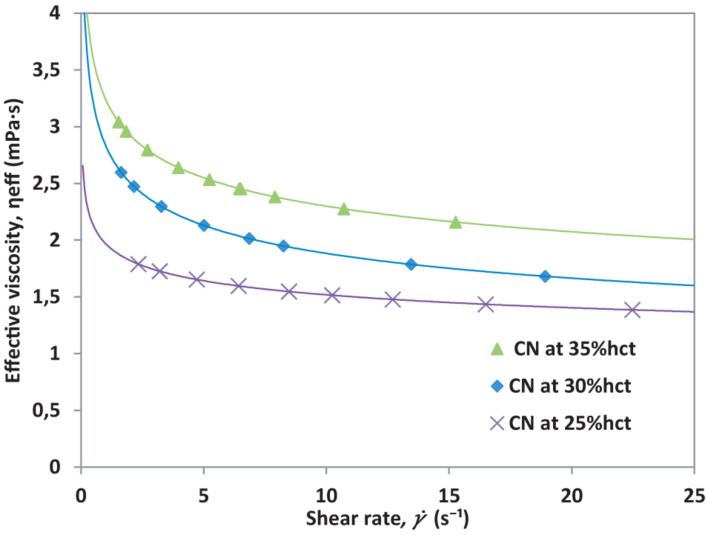
Effective viscosity vs. shear rate for different hematocrit of healthy blood from a single healthy donor (CN).

As seen in Figure 2, the shear rate, which is the normalization of the front velocity respect to the channel height, increases as the hct (%) value decreases under constant pressure conditions. Plasma has the highest front velocity when the same pressure is applied. Once the mean front velocity associated with each set pressure has been measured, it is possible to calculate the effective viscosity for the different hematocrit samples according to their plasma. The effective viscosity, η_eff_ is calculated using Eq. 3. The effective viscosity η_eff_ depends on plasma viscosity. The result is shown in Figure 3.

**FIGURE 4:**
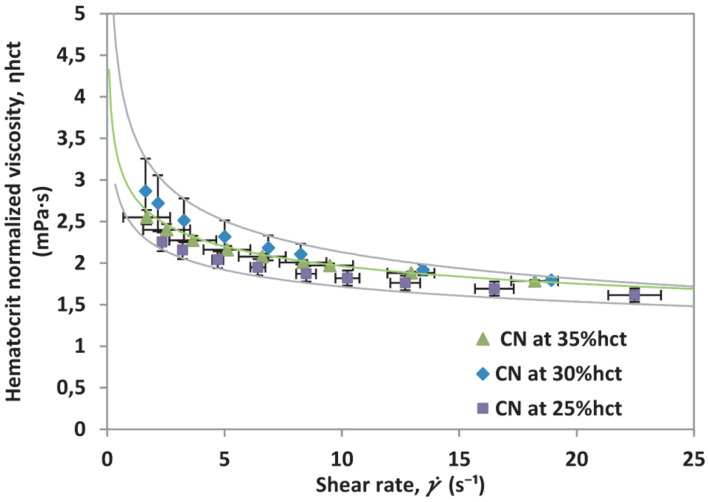
Hematocrit normalized viscosity vs. shear rate for different hematocrit percentages from a single healthy donor (CN).

The normalization of viscosity according to hematocrit can be done by applying Eq. 4, which takes the highest hematocrit from the sample measured and compares it to the reduced hematocrit samples. Since the samples come from the same donor, if the equations were properly deduced, all curves must collapse onto a single viscosity curve for all the hematocrit levels, meaning that the differences observed before are only due to hematocrit variations. The hematocrit normalized viscosity, η_hct_ is presented in Figure 4.

The results presented in Figure 4, indicate that the method employed is useful to remove effects caused by hematocrit differences. Furthermore, it will be helpful to contrast with samples that show differences related to RBCs count and abnormalities affecting the cells in suspension.

**FIGURE 5:**
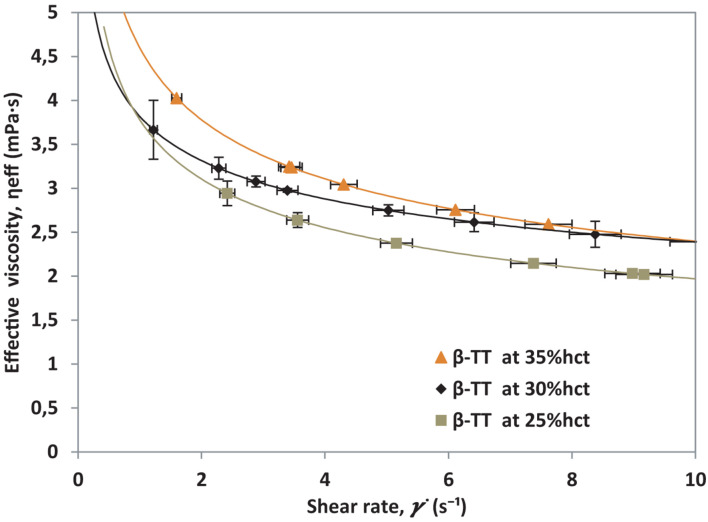
Effective viscosity vs. shear rate from a patient with β-TT at three different RBCs concentrations.

The shear rate response for each sample will depend, mostly, on the percentage of RBCs present in suspension. This response can also be affected by the presence of abnormalities developed by the blood cells, such as in β-TT. To validate the normalization of viscosity for non-healthy donors, the same experiment is conducted with β-TT samples. For validation, three hematocrit percentages (25%, 30%, and 35% hct) from a single β-TT patient are prepared. Note that β-TT samples are tested at lower $\dot{\gamma }$ to avoid hemolysis. The effective viscosity, η_eff_ is calculated for each hematocrit using Eq. 3. The resulting curves are presented in Figure 5.

**FIGURE 6:**
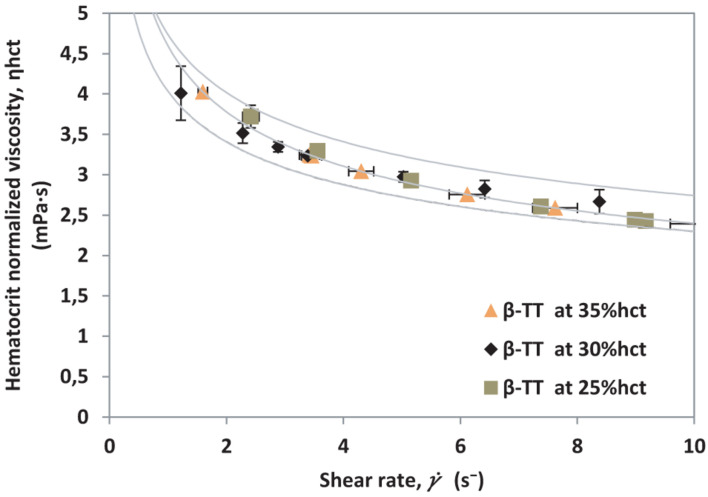
Hematocrit normalized viscosity vs. shear rate for different hematocrit percentages from a single β-TT donor.

After calculating η_eff_, hematocrit normalized viscosity, η_hct_ is calculated using Eq. 4. By taking ϕ_*max*_ = 35% hct, a normalized curve for β-TT is obtained, it is presented in Figure 6.

**FIGURE 7:**
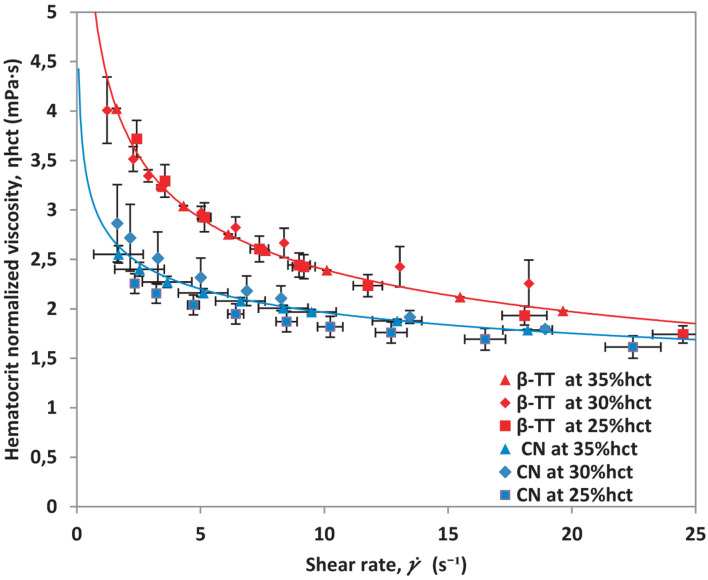
Hematocrit normalized viscosity vs. shear rate for β-TT samples and healthy blood samples.

The results obtained in Figures 4, 6 demonstrate that the method presented makes it possible to normalize different samples by hematocrit, regardless of their health condition. Using this normalization, curves for diverse pathologies could be compared without the effects of different hematocrit levels. For comparison purposes, we contrast both curves (healthy donors and β-TT donors) in Figure 7, where the measurements obtained for each patient collapse into well differentiated curves.

Moreover, the method used to measure viscosity allows us to obtain points at low shear rates, giving us the advantage to observe the shear-thinning behavior of blood. This also makes it possible to observe that viscosity values are larger at low $\dot{\gamma }$ for β-TT than for healthy samples. The hematocrit normalized viscosity curves obtained in Figure 6 for β-TT show higher viscosity than the control samples in Figure 4.

### Study of Beta-Thalassemia Trait Samples

When beta-thalassemia is present, RBC indices show microcytic anemia. Thalassemia major is characterized by reduced Hb level (<7 g/dl), mean corpuscular volume (MCV) > 50 < 70 fL and mean corpuscular hemoglobin (MCH) > 12 < 20 pg. Thalassemia intermedia is characterized by Hb levels between 7 and 10 g/dl, MCV between 50 and 80 fL, and MCH between 16 and 24 pg. Thalassemia minor (BT Trait) is characterized by reduced MCV and MCH, with increased Hb A2 level (Galanello et al., 1989; Galanello and Origa, 2010). To carry out this study, whole blood samples from healthy donors and patients diagnosed with β-TT are used. To avoid effects caused by the variation of microcytosis, the samples were classified into three groups, according to their MCV, as presented in Table 1.

**TABLE 1:** Classification of β-TT and CN samples according to their MCV.

Group	Type	MCV (fL)
Lowest	Beta-thalassemia	58–65
Very low	Beta-thalassemia	66–70
Low	Beta-thalassemia	71–75
Normal volume	Healthy blood	90–92

**FIGURE 8:**
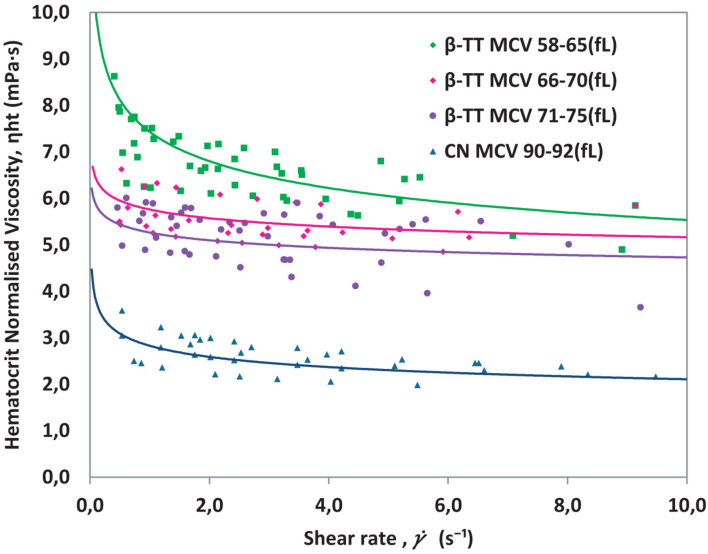
Hematocrit normalized viscosity for β-TT and healthy control blood (CN) classified by MCV in Table 1. Whole blood is used in all cases.

For each MCV group, five samples from different patients were tested. The effective viscosity was calculated with Eq. 3. The η_hct_, obtained by using Eq. 4, for all the measured samples are presented in Figure 8. A tendency line indicating the average value obtained for each kind of sample is included.

As seen in Figure 8, β-TT samples with the smallest MCV (58–65 fL) have a higher viscosity than β-TT samples with higher MCV (66–70 fL and 71–75 fL). This indicates that the size of the erythrocyte may be affecting the viscosity measurement results. When two or more samples have equivalent hct (%) and different MCV, it implies that the number of RBCs is greater as lower is the MCV, which generates a greater number of cell rows in the flow when circulating through the microchannel. This effect, studied in detail for healthy red blood cells in a previous work (Lázaro et al., 2019) implies a stronger RBC interaction which leads to an increase in the magnitude of the viscosity, similar to the effect on viscosity observed in Figure 8. The parameters *m* and *n* describing the viscosity curves, calculated using Eq. 2, for each sample included in Figure 8, are presented in Table 2.

**TABLE 2:** Average values of *m* and *n* for healthy blood and β-TT samples according to MCV in fL.

Type	Group	*m*	*n*	MCV (fL)
β-TT	Lowest	7.4295	0.872	58–65
β-TT	Very low	5.7582	0.953	66–70
β-TT	Low	5.6265	0.955	71–75
CN	Normal volume	2.8272	0.880	90–92

*Viscosity is normalized by hematocrit.*

The β-TT samples with the smallest MCV values have the highest average *m* value, which equals the value of viscosity at $\dot{\gamma }=1$. β-TT samples with MCV between 66 and 75 fL have very similar rheological parameters. The curves in Figure 8 differ from those of healthy blood for two reasons, because the MCV is different, and because the mechanical properties of healthy and pathological red blood cells are different. We want to verify that alterations such as the changes in cell morphology and rigidity caused by the presence of β-TT, have a direct effect on the viscosity. The normal reference range for MCV is typically 80–100 fL, but this value is altered in some conditions.

### Comparison of Blood Samples With Microcytic Red Blood Cells: Iron Deficiency Anemia and Beta-Thalassemia Trait

Anemia is a condition in which the number of red blood cells, and consequently their oxygen-carrying capacity, is insufficient to meet the physiologic needs of the body, and it is diagnosed as a decrease in the blood hemoglobin concentration (Hb) (Vitamin and Mineral Nutrition Information System (VMNIS), 2011). Iron deficiency limits the synthesis of heme, which in turn limits the synthesis of hemoglobin (Nagababu et al., 2008; DeLoughery, 2017), as it happens in β-TT as a result of a mutation. It is also characterized by decreased ferritin, and MCV, while total iron-binding capacity and red blood cell distribution are increased. Hypochromic red blood cells can be also be observed by visual inspection on a blood smear (Wagley, 1953; DeLoughery, 2017).

**FIGURE 9:**
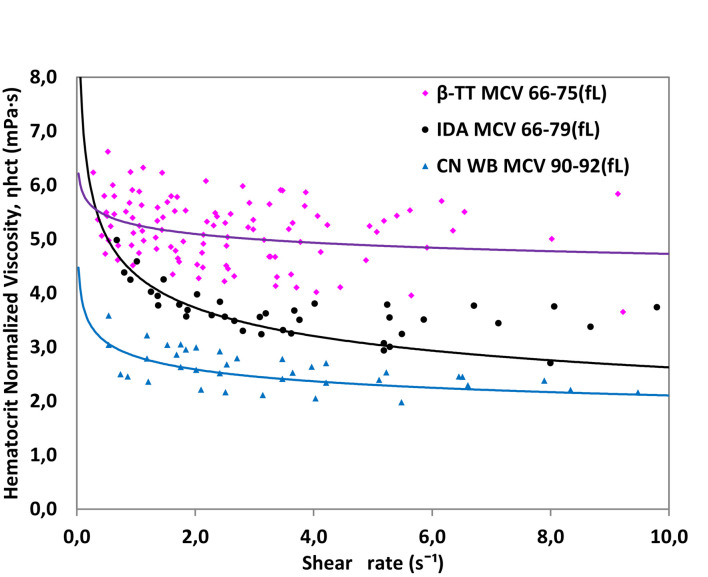
Hematocrit normalized viscosity for ten samples of β-TT, five samples of IDA and five samples from healthy donors (CN). Whole blood in all cases.

To confirm that the alterations seen in the viscosity of β-TT samples are not only attributed to microcytosis, we compared results obtained from samples with MCV of 66–75 fL to results of samples of IDA patients, with similar values of MCV. Using the same method as in Figure 8, we tested ten β-TT samples with MCV between 66 and 75 fL, five IDA samples with MCV between 66 and 79 fL and five samples from healthy donors with MCV between 90 and 92 fL. The results are shown in Figure 9.

Figure 9 shows that despite having similar MCV, β-TT and IDA samples have different viscosity patterns. The *m* and *n* parameters describing the viscosity curve for each group are presented in Table 3.

**TABLE 3:** Average values of *m* and *n* for IDA and samples according to MCV after normalizing by hematocrit.

Sample	Type	*m*	*n*	MCV fL
β-TT	Beta-thalassemia trait	5.6923	0.954	66–75
IDA	Iron deficiency anemia	4.3391	0.782	66–79
Control	Healthy blood	2.8272	0.880	90–92

Iron deficiency anemia and β-TT curves have a different viscosity pattern even though they have similar MCV, indicating that pathological RBC conditions are reflected in viscosity, and they can be differentiated with the micro-rheometer. The IDA viscosity results show a much less Newtonian behavior; the *n* value differs more from 1 than that obtained with the β-TT samples. Since both diseases present Hb deficiency and RBCs with similar MCV, we conclude that the difference observed in viscosity between the β-TT and IDA cannot be considered a consequence of differences in RBC size. The higher viscosity obtained for the β-TT samples might be attributed to a greater rigidity in the RBC membrane. This can be caused by the excess of α chains that bind to the RBC membrane (DeLoughery, 2017; Payán-Pernía et al., 2021).

### Deformability Changes in Beta-Thalassemia Trait and Iron Deficiency Anemia

**FIGURE 10:**
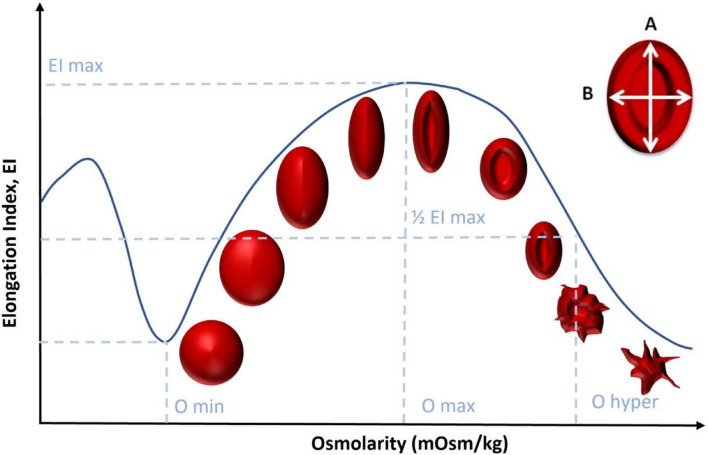
RBC elongation index evolution as the osmolarity increases using LoRRCA.

To verify that the differences in viscosity found between β-TT, IDA, and healthy donors are caused by changes in the properties of RBCs, we compare the results obtained with the LoRRCA ektacytometer between the samples. By measuring the elongation index, the LoRRCA can detect elasticity changes in single cells. The measurements performed determine elongation of a huge number of RBCs utilizing laser detection, at different osmolarity values, as illustrated in Figure 10.

The elongation index (EI) depicted in Figure 10 is determined by the expression:


(10) $$\mathrm{EI}=\frac{\text{A}-\text{B}}{\text{A}+\text{B}}$$

where A and B are the major and minor dimensions of the RBC, respectively. With this test, it is possible to obtain a characteristic profile of each sample, according to the deformability of the RBCs as a function of osmolarity. These results do not depend on the hematocrit value of the samples.

**FIGURE 11:**
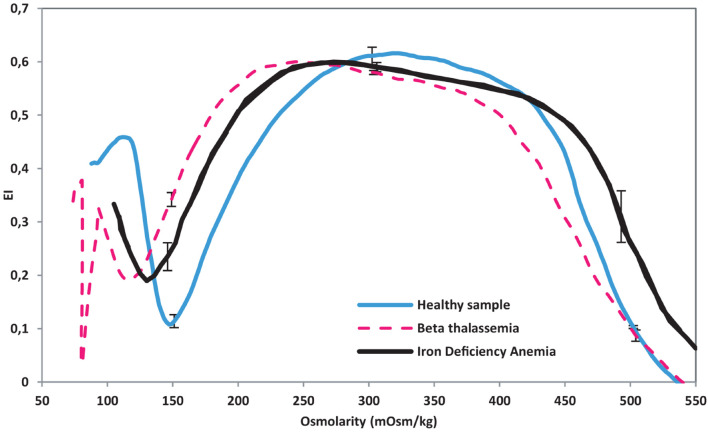
Osmoscan curve obtained from LoRRCA. Ten samples of β-TT, five samples of IDA and five samples from healthy donors were used. IDA curve adapted from Payán-Pernía et al. (2021).

Figure 11 shows the Osmoscan curve for the healthy subjects, IDA samples and β-TT samples. These results were previously described and differences between the three groups were statistically significant, reflecting deformability differences between these three states (Payán-Pernía et al., 2021). When β-TT is present, the ektacytometry curve is displaced to the left with respect to healthy RBC, revealing a decrease in the RBC deformability capacity.

The osmotic gradient ektacytometry analysis shown in Figure 11 was performed using the Osmoscan module of the LoRRca MaxSis (RR Mechatronics). By measuring elongation index, LoRRCA can detect individual erythrocyte deformability in a mixture of RBCs. When β-TT is present, the ektacytometry curve is displaced to the left with respect to healthy RBC. IDA samples, however, show a shift that put them in between β-TT and the control samples. By observing Figures 9, 11, it is possible to establish that alterations in RBC wall rigidity can be translated into viscosity changes. After normalizing the hct (%), the viscosity of samples for IDA falls below the viscosity of β-TT and over the viscosity of control samples. As seen in Figure 10, β-TT samples have a higher viscosity than control samples. This is consistent with samples that appear to have a left shift in their ektacytometry measurements. Observing the two results together lets us have a bigger picture of the effects of single cell rheology. LoRRCA is very powerful at finding individual differences within cells. The results obtained in this study, demonstrate that our method can detect the presence of these alterations through the study of the fluid front rheology of the whole blood. Therefore, the method proposed can distinguish differences between healthy blood and blood affected by pathologies that modify the red blood cell rheology. Changes in viscosity seem to have a direct relation to individual cells’ changes in β-TT.

## Conclusion

Through this study, we have seen that the micro-rheometer presented here has great potential as a diagnostic tool for hematological diseases. This has been demonstrated by differentiating three sets of samples, from healthy donors, β-TT and IDA patients. Using a microfluidic method we have been able to distinguish between samples with different RBCs abnormalities, despite the fact that the donors of each set have different ages and distinct hct (%). Comparison with a standardized red blood cell elasticity characterization method, the LoRRCA ektacytometer, has allowed us to verify that our findings were correct.

The sample characterization techniques used in this paper are based on the use of normalized curves, differentiated for distinct pathologies. Using a mathematical method for hematocrit normalization (Trejo-Soto and Hernández-Machado, 2021), a single viscosity curve is calculated for healthy donors. Using the same method, a normalized viscosity curve of β-TT is obtained. The hematocrit normalized viscosity η_hct_, does not contain the effects caused by hematocrit levels. This was made to perform an adequate comparison between samples from the same or different patients, both in healthy and non-healthy conditions, independent of their hct (%). Thanks to this method, we obtained curves with clearly differentiated parameters for healthy blood, β-TT, and IDA.

The experimental results herein obtained, show that the MCV seems to affect the viscosity of the samples. Samples with distinct MCV did not follow the normalized curve path. Once the samples were segregated into three groups based on their MCV, a clear pattern showed up. As a general observation, lower MCV accounted for higher viscosity values. For this motive, it is reasonable to do a comparison between β-TT and IDA samples with similar MCV only. It was found, however, that even at comparable MCV and after hematocrit normalization, β-TT has a higher viscosity than both IDA and control samples. This difference in viscosity might be caused by differences in the affected deformation capability of RBCs and the membrane changes caused by the α chains disorders that characterize β-TT (Payán-Pernía et al., 2021).

Reasoning on the basis that high blood viscosity might be caused by changes in the RBC wall rigidity, it is plausible to infer that these differences may be reflected in an ektacytometry analysis. It was found that the osmotic gradient curve obtained by LoRRCA for IDA samples is shifted to the left of the curve for healthy donors and to the right of the curve for β-TT patients, reflecting that the rigidity of RBCs in IDA samples is higher than that of healthy donors, and at the same time smaller than the rigidity of RBCs in β-TT samples. This is compatible with the viscosity results in which IDA samples have a lower viscosity than β-TT patients and higher than healthy donors. The differences observed in viscosity between the samples analyzed could be a result of changes in the rigidity of the RBCs when β-TT is present (Payán-Pernía et al., 2021).

The general conclusion of this work is that the micro-rheometer is a promising tool for early detection of different blood diseases can differentiate between two types of blood diseases, β-TT and IDA, regardless their hematocrit level and the presence of microcytosis. It has been also demonstrated that blood viscosity is an accurate indicator of the blood health condition, especially when the pathology in study targets the RBC and affects its shape, deformation capacity and size. Furthermore, the samples show a clear difference when compared to healthy samples, by using the microrheology technique described. A correct characterization on blood has been made through an adequate setup, without the need of complex equipment and with just a small sample. At the same time, the results obtained are comparable to those delivered by standard diagnostic techniques, such as LoRRCA, which is the gold standard method for ektacytometry evaluation of RBCs. The results obtained are important for the development of a functional point-of-care diagnosis device for the fast and effective detection of hemolytic anemias such as IDA and β-TT. PoC devices can provide a very positive outcome for populations located in remote areas of the world where the access to resources is limited. Diagnostic tools that provide fast and reliable results constitute an easy way to keep the follow up of patients up to date, without the need of commuting and the use of hospital facilities. In the near future, a cheap, easy to use, and small device can be used for both triage in hospitals and at home by patients suffering of RBC altering conditions such IDA and β-TT.

## Data Availability Statement

The original contributions presented in the study are included in the article/supplementary material, further inquiries can be directed to the corresponding author/s.

## Ethics Statement

The studies involving human participants were reviewed and approved by the Ethics Committee of IJC [Comité Ético de Investigación Clínica (CEIC)] and the Bioethics Committee of the University of Barcelona (IRB 00003099). The patients/participants provided their written informed consent to participate in this study.

## Author Contributions

AH-M and J-LV-C conceived the study. LM-M and MC-F performed the experiments, processed the data, and wrote the manuscript. CR-L and JF-T helped to interpret the results. EK collected and analyzed the samples prepared by IH-R and CM-I. SP-P, TA, AH-M, MC-F, and CT-S supervised the research work and revised the results and discussion. All authors contributed to the manuscript revision, read, and approved the submitted version.

## Conflict of Interest

The authors declare that the research was conducted in the absence of any commercial or financial relationships that could be construed as a potential conflict of interest. The handling editor declared a past collaboration with one of the author, J-LV-C.

## Publisher’s Note

All claims expressed in this article are solely those of the authors and do not necessarily represent those of their affiliated organizations, or those of the publisher, the editors and the reviewers. Any product that may be evaluated in this article, or claim that may be made by its manufacturer, is not guaranteed or endorsed by the publisher.
